# Socioeconomic Inequities in Vaccine Hesitancy Among Native Hawaiians and Pacific Islanders

**DOI:** 10.1089/heq.2022.0033

**Published:** 2022-08-23

**Authors:** Raynald A. Samoa, Lan N. Ðoàn, Anne Saw, Nia Aitaoto, David Takeuchi

**Affiliations:** ^1^ Department of Clinical Diabetes, Endocrinology and Metabolism, Diabetes and Metabolism Research Institute, City of Hope, Duarte, California, USA.; ^2^ Department of Population Health Section for Health Equity, NYU Grossman School of Medicine, New York, New York, USA.; ^3^ Department of Psychology, DePaul University, Chicago, Illinois, USA.; ^4^ Department of Nutrition & Integrative Physiology, College of Health, University of Utah, Salt Lake City, Utah, USA.; ^5^ School of Social Work, University of Washington, Seattle, Washington, USA.

**Keywords:** Native Hawaiian, Pacific Islanders, NH/PI, COVID-19, vaccine hesitancy

## Abstract

**Purpose:**

COVID-19 vaccine hesitancy exists in communities of color who are disproportionately impacted by COVID-19. In many states, Native Hawaiians and Pacific Islanders (NHs/PIs) experience the highest rates of COVID-19 confirmed cases and mortality among U.S. ethnic/racial groups. National trends regarding vaccine hesitancy among NHs/PIs are currently lacking.

**Methods:**

Data were derived from the Asian American and NH/PI COVID-19 Needs Assessment Project, a national survey conducted during January–April 2021. The final analytic sample included 868 NH/PI adults. Logistic regression analyses were conducted to estimate odds ratios for vaccine hesitancy.

**Results:**

Vaccine hesitancy ranged from 23% among Other PIs to 56.3% among Tongan adults. Younger adults (18–24 and 25–44 years), those with lower educational attainment, and those with lower income were more vaccine hesitant. Overall, education and income showed a strong association with vaccine hesitancy in bivariate logistic models. However, the associations between vaccine hesitancy and education and income varied by NH/PI groups. NHs, Samoans, and Multiethnic NHs/PIs showed the most consistent associations between the socioeconomic position variables and vaccine hesitancy.

**Conclusions:**

The examination of vaccine hesitancy among NHs/PIs follows the socioeconomic gradient for some ethnic groups but not others. More studies are needed to determine what other socioeconomic indicators may be associated with health among specific NH/PI ethnic groups.

**Policy Implications:**

Reforms are needed to overcome structural racism underlying NH/PI evidence production, which currently renders NHs/PIs invisible. Innovative solutions based on successful community efforts can help deconstruct racist data inequities experienced by NHs/PIs.

## Introduction

A disturbing trend in COVID-19 cases and death rates has been observed among Native Hawaiian and Pacific Islander (NH/PI) communities throughout the pandemic. As of January 27, 2022, data from the NH/PI COVID-19 Data Policy Lab Dashboard show that NHs/PIs have the highest positive case rates than any other racial/ethnic group in 15 of the 21 states that disaggregate NH/PI data.^1^ In the 16 states that report disaggregated mortality data, NHs/PIs have experienced the highest mortality rates due to COVID-19 in 13 of these states.^1^

Released in December 2021, a safe, effective, comprehensive vaccination campaign has been front and center in the White House strategy, to control the spread of COVID-19. The specific intent of this national plan is to maximize the vaccination of highly disproportionately impacted populations such as communities of color.^2^ Yet despite the proven effectiveness of mRNA-1273,^3^ BNT162b2,^4^ and JNJ-78436735^5^ formulations of the SARS-CoV-2 vaccines and the relatively low risk of adverse events with their use, notable vaccine hesitancy has been reported in vulnerable communities.^3–5^

Willis et al. revealed higher vaccine hesitancy in Hispanic and African American populations.^6^ In multivariate analyses, vaccine hesitancy was significantly associated with demographic variables including socioeconomic position (SEP) such as gender, education, employment, income, and having children at home.^7^

National and local media have reported on vaccine hesitancy in NH/PI communities, but empirical investigations are lacking. NHs/PIs are frequently missing from analyses of vaccine hesitancy, are lumped together with Asian Americans, or, when studies include NHs/PIs, the small sample size prohibits in-depth, meaningful statistical analyses. It is critical to examine the heterogeneity of the NH/PI population to move beyond the confinements of the broad racial category.

The social determinants of health (SDOH) paradigm is a useful framework to begin an analysis of the social factors associated with vaccine hesitancy among NH/PI ethnic groups.^8^ SDOH focuses attention on the conditions of life outside of the medical system that influencse individual and group differences in health status and health care. Living, work, school, and recreational conditions such as the unequal distribution of income, education, wealth, parks, good paying jobs, access to safe neighborhoods rather than individual factors such as genetics and lifestyles are more powerful explanations of disease and premature deaths.

In this study, we examine how education and income, as measures of SEP, are associated with vaccine hesitancy. Although education and income are generally accepted correlates of health, it is more established for Whites than for other racial and ethnic groups (e.g., Blacks and Latinx communities). It certainly has not been established for NHs/PIs.^9^^(pp310–352),^^10^Accordingly, this article examines how education and income are tied to vaccine hesitancy for specific NH/PI ethnic groups. Education and income are usual measures of SEP and are linked to knowledge, skills, reasoning, and access to personal and professional networks that promote healthier lifestyles.

It should be noted that factors other than education and income may have also influenced the vaccine hesitancy of respondents such as the high prevalence of uninsured and underinsured NHs/PIs and the mistrust and hesitancy of NHs/PIs in seeking health care services. Furthermore, preliminary results from the survey suggest a delay in seeing one's medical provider reported in NHs/PIs regardless of education level.

The analyses that follow provide the means to examine whether SEP is significant overall for NHs/PIs and whether the associations between SEP and vaccine hesitancy are consistent across specific NH/PI ethnic groups.

## Methods

### Survey

The Asian American and NH/PI COVID-19 Needs Assessment was conducted between January 19 and April 9, 2021, as part of a larger study examining the impact of COVID-19 on communities of color.^11^ The needs assessment consisted of questions regarding physical health, mental health, stress and coping experiences, impact of racism, educational challenges, food security, housing security, labor and economics, access to health care and health information, including COVID-19 testing, and community supports and assets. Survey respondents were recruited through a Qualtrics online panel and convenience samples through community outreach.

Eligible criteria for study participation included age 18 years or older, identification as Asian American and/or NH/PI, and residing in the United States since March 13, 2020. The survey was administered in four NH/PI languages (Samoan, Tongan, CHamoru, and Marshallese), English, and eight Asian languages. This study was approved by the Asian American Pacific Community Health Organizations (AAPCHO) institutional review board.

To promote equitable data collection for NH/PI populations who have been inequitably represented in research, the research team employed several strategies within the study design. First, investigators enlisted cultural protocols in every aspect of the study design. Leveraging long-standing relationships, a core group of community-based organizations (CBOs) and community advocates representing six distinct geographical regions in the United States was assembled into the Pacific Islander COVID-19 Response Team (PICRT)^12^ and served on an expert panel that informed survey categories, formulated and approved all survey questions, vetted all data collection methods to ensure cultural appropriateness, and optimized survey responsiveness (e.g., collecting data at vaccination drives, and distributing surveys along with food distribution for older and homebound individuals).

Second, the largest NH/PI ethnic groups were oversampled to mitigate the problem of small sample sizes, disallowing disaggregation. Third, the survey instrument was translated into four PI languages. Fourth, we focused recruitment efforts on geographic regions with large concentrations of target NH/PI groups with respective regional community leads of the PICRT vetting the feasibility of obtaining adequate number of responses for each selected region.

This study focuses specifically on respondents who self-identified as NHs/PIs (*n*=1262). Respondents were excluded from analyses if there were missing responses for vaccine hesitancy (main outcome) and demographic variables including NH/PI ethnicity, gender, education, and income. The final analytic sample was 868 respondents.

### Main outcome

Respondents were asked, “How likely are you to get vaccinated for COVID-19 once a vaccination is available to the public?” and responses were very unlikely, somewhat unlikely, somewhat likely, very likely, and unsure. Individuals who responded very unlikely, somewhat unlikely, or unsure were categorized as *hesitant* to getting the COVID-19 vaccine. Individuals who responded very likely or somewhat likely were categorized as *not hesitant*.

### SEP and sociodemographic variables

The two SEP variables of interest include education and income. Education level was categorized as less than high school/completed high school graduate or general educational development (GED), some college, Associate in Arts (AA) or technical degree, bachelor's degree, and graduate degree or higher. Annual household income was categorized as <$25,000, $25,000 to <$50,000, $50,000 to <$75,000, $75,000 to <$100,000, and $100,000 or more. The survey also collected self-reported sociodemographic characteristics such as age (18–24 years, 25–44 years, 45–64 years, 65 years or older) and gender (male, female, transgender/nonbinary/other).

There were six NH/PI ethnic group categories: NH, Samoan, Tongan, Marshallese, Other PI (respondents who identified as Other PI, Fijian, CHamoru, or Chuukese), and Multiethnic NHs/PIs (respondents who identified with more than one NH/PI ethnic group). Owing to the small number of respondents for Other PI, Fijian, CHamoru, or Chuukese, all were grouped into Other PIs.

### Statistical analysis

We calculated frequencies for vaccine hesitancy and sociodemographic variables for the overall NH/PI sample. We conducted binary logistic regression to estimate odds ratios (ORs) and 95% confidence intervals (CIs) for the association between vaccine hesitancy and the SEP and sociodemographic variables. We then examined the SEP and sociodemographic variables within each of the large NH/PI ethnic groups. All data were analyzed using R Studio Version 1.4.1106 “Tiger Daylily.”^13^

## Results

Table 1 summarizes the demographic characteristics for the overall NH/PI sample and stratified by vaccine hesitancy. Almost one-third of the overall sample self-identified as Samoan (31.7%), followed by Tongan (18.2%), NH (16.1%), Other PI (14.1%), Multiethnic (11.3%), and Marshallese (8.6%). More than half of respondents were 25–44 years old (55.2%) and female (65%). Education and income were skewed toward the lower end of the distribution (Supplementary Table S1).

**Table 1.** Demographic Characteristics for the Overall Sample and by Hesitancy

	Overall		Not hesitant		Hesitant	
	***n***	%	***N***	% Not hesitant	***n***	% Hesitant
Total	868		543		325	
NH/PI subgroup						
Native Hawaiian	140	16.1	107	76.4	33	23.6
Samoan	275	31.7	178	64.7	97	35.3
Tongan	158	18.2	69	43.7	89	56.3
Marshallese	75	8.6	40	53.3	35	46.7
Other Pacific Islander	122	14.1	94	77.0	28	23.0
Multiethnic	98	11.3	55	56.1	43	43.9
Age						
18–24 years	138	15.9	84	60.9	54	39.1
25–44 years	479	55.2	276	57.6	203	42.4
45–64 years	214	24.7	155	72.4	59	27.6
65+ years	37	4.3	28	75.7	9	24.3
Gender						
Male	288	33.2	177	61.5	111	38.5
Female	564	65.0	353	62.6	211	37.4
Transgender/nonbinary/other	16	1.8	13	81.3	3	18.8
Education						
Less than HS, completed HS or GED	245	28.2	121	49.4	124	50.6
Some college	227	26.2	136	59.9	91	40.1
AA or technical degree	148	17.1	89	60.1	59	39.9
Bachelor's degree	158	18.2	121	76.6	37	23.4
Graduate degree	90	10.4	76	84.4	14	15.6
Income						
Less than $25,000	191	22.0	101	52.9	90	47.1
$25,000 to <$50,000	231	26.6	135	58.4	96	41.6
$50,000 to <$75,000	190	21.9	111	58.4	79	41.6
$75,000 to <$100,000	106	12.2	79	74.5	27	25.5
$100,000 or more	150	17.3	117	78.0	33	22.0

AA, associate in arts; HS, high school; GED, general educational development.

Vaccine hesitancy ranged from 23.0% among Other PI to 56.3% among Tongan adults. Vaccine-hesitant respondents on average were younger, with 39.1% of adults 18–24 years and 42.4% of adults 25–44 years old who were vaccine hesitant. Vaccine hesitancy was 50.6% among respondents with a high school or GED level education or less; but there was lower hesitancy among respondents with some college, AA or technical degree, bachelor's degree, or graduate degree. For annual household income, vaccine hesitancy was 47.1% among respondents who earned <$25,000; this percentage decreased to 22% among respondents who earned $100,000 or more.

Table 2 assessed the binary relationship between COVID-19 hesitancy and our demographic variables. Compared with NH respondents, Samoan (OR=1.77; 95% CI=1.12–2.83), Tongan (OR=4.18; 95% CI=2.55–6.97), Marshallese (OR=2.84; 95% CI=1.56–5.19), and Multiethnic (OR=2.53; 95% CI=1.46–4.46) respondents were significantly more likely to be vaccine hesitant.

**Table 2.** Binary Logistic Regression of COVID-19 Hesitancy by Demographic Variables

Independent variable	OR	95% CI	***p***-Value
NH/PI subgroup			
Native Hawaiian	1.00 (Ref)		
Samoan	**1.77**	**1.12–2.83**	**0.02**
Tongan	**4.18**	**2.55–6.97**	**<0.001**
Marshallese	**2.84**	**1.56–5.19**	**<0.001**
Other Pacific Islander	0.97	0.54–1.71	0.91
Multiethnic	**2.53**	**1.46–4.46**	**<0.01**
Age			
18–24 years	1.00 (Ref)		
25–44 years	1.14	0.78–1.69	0.50
45 years or older	**0.58**	**0.37–0.90**	**0.02**
Gender			
Male	1.00 (Ref)		
Female	0.99	0.76–1.29	0.94
Transgender/nonbinary/other	0.75	0.30–1.90	0.55
Education			
Less than HS, completed HS or GED	1.00 (Ref)		
Some college	**0.65**	**0.45–0.94**	**0.02**
AA or technical degree	**0.65**	**0.43–0.98**	**0.04**
Bachelor's degree	**0.30**	**0.19–0.46**	**<0.001**
Graduate degree	**0.18**	**0.09–0.33**	**<0.001**
Income			
Less than $25,000	1.00 (Ref)		
$25,000 to <$50,000	0.80	0.54–1.17	0.25
$50,000 to <$75,000	0.80	0.53–1.20	0.28
$75,000 to <$100,000	**0.38**	**0.22–0.64**	**<0.001**
$100,000 or more	**0.32**	**0.19–0.51**	**<0.001**

Bold value indicates statistically significant *p*-value <0.05.

CI, confidence interval; OR, odds ratio.

Respondents who were older (45 years or older) (OR=0.58; 95% CI=0.37–0.90); had some college (OR=0.65; 95% CI=0.45–0.94), AA or technical (OR=0.65; 95% CI=0.43–0.98), bachelor's (OR=0.30; 95% CI=0.19–0.46), or graduate (OR=0.18; 95% CI=0.09–0.33) degree; and earned $75,000 to <$100,000 (OR=0.38; 95% CI=0.22–0.64) or $100,000 or more (OR=0.32; 95% CI=0.19–0.51) were less likely to be vaccine hesitant than respondents who were 18–24 years old, who had less than a high school, high school, or GED education, and who earned <$25,000, respectively.

Table 3 gives the adjusted logistic regression models for age, gender, and education, stratified by NH/PI ethnicity. Among NH respondents, female respondents (OR=3.85; 95% CI=1.33–11.19) were more vaccine hesitant than their male counterparts and respondents with a graduate degree (OR=0.06; 95% CI=0.01–0.57) were less vaccine hesitant than respondents with less than a high school, high school, or GED education. For Samoan respondents, respondents with a bachelor's degree (OR=0.39; 95% CI=0.16–0.92) or graduate degree (OR=0.39; 95% CI=0.15–0.97) were less vaccine hesitant than respondents with less than a high school, high school, or GED education.

**Table 3.** Adjusted Logistic Regression Models of COVID-19 Hesitancy with Education

	Native Hawaiian (***n***=131)		Samoan (***n***=262)		Tongan (***n***=155)		Marshallese (***n***=71)		Other PI (***n***=117)		Multiethnic (***n***=93)	
	OR (95% CIs)	***p-***Value	OR (95% CIs)	***p-***Value	OR (95% CIs)	***p-***Value	OR (95% CIs)	***p-***Value	OR (95% CIs)	***p-***Value	OR (95% CIs)	***p-***Value
Age												
18–24 years	1.00 (Ref)		1.00 (Ref)		1.00 (Ref)		1.00 (Ref)		1.00 (Ref)		1.00 (Ref)	
25–44 years	1.93 (0.62–5.97)	0.25	0.83 (0.41–1.69)	0.60	2.03 (0.55–7.44)	0.29	0.38 (0.07–2.18)	0.28	2.20 (0.52–9.36)	0.29	2.32 (0.69–7.77)	0.17
45 years or older	1.29 (0.40–4.20)	0.67	0.51 (0.24–1.11)	0.09	0.69 (0.17–2.74)	0.59	**0.10 (0.01–0.81)**	**0.03**	0.98 (0.21–4.52)	0.98	2.60 (0.58–11.64)	0.21
Gender												
Male	1.00 (Ref)		1.00 (Ref)		1.00 (Ref)		1.00 (Ref)		1.00 (Ref)		1.00 (Ref)	
Female	**3.85 (1.33–11.19)**	**0.01**	1.13 (0.64–1.99)	0.68	0.60 (0.29–1.23)	0.16	0.49 (0.16–1.52)	0.22	0.98 (0.39–2.44)	0.96	1.97 (0.66–5.88)	0.22
Transgender/nonbinary/other	2.55 (0.21–31.16)	0.46	0.71 (0.12–4.06)	0.70					—	—	—	—
Education												
Less than HS, completed HS or GED	1.00 (Ref)		1.00 (Ref)		1.00 (Ref)		1.00 (Ref)		1.00 (Ref)		1.00 (Ref)	
Some college	0.56 (0.17–1.77)	0.32	0.60 (0.30–1.20)	0.15	0.85 (0.36–1.97)	0.70	2.41 (0.68–8.58)	0.17	0.71 (0.20–2.58)	0.61	0.32 (0.10–1.05)	0.06
AA or technical degree	1.12 (0.34–3.78)	0.85	0.87 (0.40–1.91)	0.73	0.52 (0.20–1.37)	0.18	0.91 (0.19–4.28)	0.91	0.63 (0.17–2.31)	0.49	0.38 (0.08–1.84)	0.23
Bachelor's degree	0.25 (0.06–1.03)	0.05	**0.39 (0.16–0.92)**	**0.03**	**0.21 (0.08–0.59)**	**<0.01**	0.38 (0.04–3.86)	0.41	0.37 (0.10–1.36)	0.14	**0.18 (0.05–0.73)**	**0.02**
Graduate degree	**0.06 (0.01–0.57)**	**0.01**	**0.39 (0.15–0.97)**	**0.04**	—	—			0.42 (0.09–1.98)	0.27	**0.02 (0.00–0.22)**	**<0.01**

Bold value indicates statistically significant *p*-value < 0.05.

Among Tongan respondents, respondents with a bachelor's degree (OR=0.21; 95% CI=0.08–0.59) were less vaccine hesitant than those with less than a high school, high school, or GED education. Marshallese respondents who were 45 years or older (OR=0.10; 95% CI=0.01–0.81) were less vaccine hesitant than adults 18–24 years old; no statistical association was observed between vaccine hesitancy and education after controlling for age and gender. Multiethnic respondents with a bachelor's degree (OR=0.18; 95% CI=0.05–0.73) and graduate degree (OR=0.02; 95% CI=0.00–0.22) were less vaccine hesitant than respondents with less than a high school, high school, or GED education. There was no statistical association observed between vaccine hesitancy and education for Other PI adults.

Table 4 gives the adjusted logistic regression models for age, gender, and income. Among NH respondents, female respondents (OR=3.16; 95% CI=1.07–9.38) were more vaccine hesitant than their counterparts; adults who earned $50,000 to < $75,000 (OR=0.26; 95% CI=0.07–0.99) and $100,000 or more (OR=0.07; 95% CI=0.01–0.67) were less vaccine hesitant than respondents earning <$25,000. When controlling for age and gender, Samoan respondents who earned $25,000 to <$50,000 (OR=0.45; 95% CI=0.22–0.94), $75,000 to <$100,000 (OR=0.25; 95% CI=0.09–0.68), and $100,000 or more (OR=0.31; 95% CI=0.14–0.68) were less vaccine hesitant than respondents earning <$25,000.

**Table 4.** Adjusted Logistic Regression Models of COVID-19 Hesitancy with Income

	Native Hawaiian (***n***=131)		Samoan (***n***=262)		Tongan (***n***=155)		Marshallese (***n***=71)		Other PI (***n***=117)		Multiethnic (***n***=93)	
	OR (95% CIs)	***p-***Value	OR (95% CIs)	***p-***Value	OR (95% CIs)	***p-***Value	OR (95% CIs)	***p-***Value	OR (95% CIs)	***p-***Value	OR (95% CIs)	***p-***Value
Age												
18–24 years	1.00 (Ref)		1.00 (Ref)		1.00 (Ref)		1.00 (Ref)		1.00 (Ref)		1.00 (Ref)	
25–44 years	1.90 (0.61–5.93)	0.27	0.77 (0.40–1.51)	0.45	1.45 (0.40–5.30)	0.57	0.38 (0.06–2.22)	0.28	2.61 (0.64–10.66)	0.18	3.08 (0.85–11.17)	0.08
45 years or older	1.33 (0.40–4.38)	0.64	0.61 (0.28–1.33)	0.21	0.59 (0.14–2.44)	0.47	**0.11 (0.01–0.86)**	**0.04**	1.17 (0.26–5.18)	0.84	3.09 (0.64–14.09)	0.16
Gender												
Male	1.00 (Ref)		1.00 (Ref)		1.00 (Ref)		1.00 (Ref)		1.00 (Ref)		1.00 (Ref)	
Female	**3.16 (1.07**–**9.38)**	**0.04**	0.94 (0.53–1.67)	0.83	0.64 (0.32–1.28)	0.21	0.58 (0.20–1.65)	0.31	0.93 (0.37–2.33)	0.87	0.95 (0.32–2.82)	0.93
Transgender/nonbinary/ other	1.54 (0.13–18.01)	0.73	0.50 (0.08–2.92)	0.44					—	—	—	—
Income												
Less than $25,000	1.00 (Ref)		1.00 (Ref)		1.00 (Ref)		1.00 (Ref)		1.00 (Ref)		1.00 (Ref)	
$25,000 to <$50,000	0.89 (0.31–2.57)	0.83	**0.45 (0.22–0.94)**	**0.03**	1.01 (0.27–3.73)	0.99	1.12 (0.38–3.32)	0.84	1.67 (0.53–5.26)	0.38	**0.18 (0.04–0.80)**	**0.02**
$50,000 to <$75,000	**0.26 (0.07–0.99)**	**0.05**	0.56 (0.25––1.25)	0.16	0.97 (0.27–3.49)	0.96	0.65 (0.12–3.43)	0.61	0.34 (0.08–1.42)	0.14	0.52 (0.11–2.31)	0.39
$75,000 to <$100,000	0.56 (0.15–2.09)	0.39	**0.25 (0.09–0.68)**	**<0.01**	0.48 (0.11–2.06)	0.32	1.17 (0.06–21.47)	0.92	0.95 (0.21–4.31)	0.95	**0.04 (0.01**–**0.34)**	**<0.01**
$100,000 or more	**0.07 (0.01–0.67)**	**0.02**	**0.31 (0.14–0.68)**	**<0.01**	0.87 (0.20–3.88)	0.86	—	—	0.45 (0.11–1.89)	0.28	**0.02 (0.00**–**0.21)**	**<0.01**

Bold value indicates statistically significant *p*-value < 0.05.

When controlling for gender and income, Marshallese respondents who were 45 years or older (OR=0.11; 95% CI=0.01–0.86) were less vaccine hesitant than adults 18–24 years old. Multiethnic respondents who earned $25,000 to <$50,000 (OR=0.18; 95% CI=0.04–0.80), $75,000 to <$100,000 (OR=0.04; 95% CI=0.01–0.34), and $100,000 or more (OR=0.02; 95% CI=0.00–0.21) were less vaccine hesitant than their counterparts earning <$25,000. There was no statistical difference among vaccine hesitancy and income for Tongan, Marshallese, and Other PI adults, when controlling for age and gender.

## Implications for Health Equity

Income and education show a pattern with vaccine hesitancy that is fairly similar to empirical studies on different health outcomes. People in lower SEP tend to be more hesitant to receiving the COVID-19 vaccine. If we simply accepted these findings, this study would confirm the conventional findings that SEP has a strong and stable association for ethnic groups not often typically studied. However, the acceptance of this pattern would be an error. When we examine SEP within NH/PI groups, we do not find the consistent pattern across the groups. NHs, Samoans, and Multiethnic NHs/PIs are more likely to show a pattern consistent where lower SEP respondents had higher levels of vaccine hesitancy.

Tongan, Marshallese, and Other PIs did not have consistent or statistical associations between the SEP measures and vaccine hesitancy. It is possible that larger sample sizes for these three ethnic groups would show the same association between SEP and vaccine hesitancy as it did for NHs, Samoans, and Multiethnic groups. It is also possible that a larger sample size could show the same pattern as found in these analyses. Without more research and larger samples, we can only speculate. What we do know is that these disparate findings reveal that we err in assuming that “one size fits all” in addressing health issues in NH/PI communities. Ethnicity is only one factor that shapes health outcomes in NH/PI communities.

One example would be in the vaccine hesitancy of transgender/nonbinary/other respondents. Their small sample size does not permit any possible inferences to be made, but the OR of 0.75 (95% CI=0.30–1.90) of the group would suggest a potential NH/PI subgroup whose behaviors may not entirely by reflected in the larger population (Table 2). The examination of different demographic, social, cultural, and geographic factors that are associated with health outcomes will go farther in addressing the policies and reforms that will best benefit NHs/PIs.

Our study findings highlight the syndemic nature of the current pandemic.^14^ A syndemic perspective acknowledges the biosocial complex that views disease and the social and environmental factors that promote and enhance the negative effects of disease interaction as a systemic entity requiring intervention at these upstream levels to limit further prolongation of the harmful effects of disease in populations left vulnerable by these social and environmental disease promoters.

In the case of the COVID-19 pandemic, a syndemic perspective provides a deeper understanding of how historical factors such as economic insecurity, poor educational access, and poor investment in infrastructure to support NH/PI health can place NH/PI communities at greater risk for a multitude of COVID-19-era challenges, including increased COVID-19 exposure, and, simultaneously, helps to explain why vaccine hesitancy remains high despite increased risks of exposure. A syndemic perspective can also help identify effective upstream interventions for NH/PIs, but a requisite first step is the gathering of relevant disaggregated data, particularly SEP factors.^15,16^

National educational statistics drawn from the 2019 American Community Survey (ACS) recently included disaggregated data for PIs, NHs, Samoans, and Tongans. The disaggregated percentage of NHs/PIs who have a high school diploma or GED equivalent (35.4–38%) exceeds the rate of the general U.S. population (27.1%).^17^ Yet, the percentage of NHs/PIs (16–19.2%) who report having a bachelor's degree or higher is far lower than the general population (31.5%).

This urges a deeper examination of the obstacles faced by NH/PI students in postsecondary education that would require the reporting of disaggregated data on the adequacy of financial aid, availability of scholarships, the downstream effects of the lack of intergenerational wealth, and the impact of impending educational debt along with other structural factors such as the availability of a curriculum that provides cultural context that can perhaps limit NH/PI students from completing their undergraduate studies.

NH/PI income data from the 2019 ACS 1-year estimates demonstrate disparities in median household income, poverty, and unemployment rates compared with non-Hispanic White households. But data disaggregated for NH/PI ethnic groups for these outcomes regionally are inconsistently available. Contextual details are missing that would enhance the understanding of the current economic state of NH/PI communities such as the number of individuals in a household for a reported income level and household income adjusted for cost of living in areas that would affect NHs/PIs as many live in densely populated urban regions known to have a higher cost of living such as California and Hawaii.

### Strengths and limitations

Some strengths of this project are the use of cultural protocols across all research stages and targeted recruitment of participants through a core group CBOs and community advocates nationally.

To note, the total sample for this survey was 1262 individuals, but due to missing responses for vaccine hesitancy, NH/PI ethnicity, gender, education, and income, our analytic sample was 868 and may be biased toward individuals who may be more likely to report SEP. For example, individuals with low SEP may not have completed information on education and income, so our estimate may not reflect lower SEP individuals who may have greater vaccine hesitancy.

Despite the small sample sizes for some PI ethnic groups (Other PI, Fijian, CHamoru, or Chuukese) who had to be combined into an Other PI category, our community-focused recruitment allowed for a large, ethnically, and linguistically diverse sample of NH/PI adults. It also should be noted that some of our results should be interpreted with caution because of the large CIs from our analyses. However, our findings are consistent with what we had learned from our community partners and reports from other communities of color regarding vaccine hesitancy.

To our knowledge, our findings represent the largest cohort of NHs/PIs (*n*=1262) describing their experience during the COVID-19 pandemic driven largely by the significant involvement of community organization leaders to vet the cultural value, relativity, and feasibility of all aspects of the study. The persistent presence of NH/PI community leaders to hold officials federal, state, and local officials accountable to provide data on NH/PI populations and their pivotal role in our survey would suggest that they are crucial stakeholders in these endeavors. This experience emphasizes the importance of involving communities in the identification and development of strategies to rectify the current health inequities experienced by NH/PI communities.

## Conclusion

Providing disaggregated socioeconomic data for NH/PI communities is a crucial linchpin in dismantling systemic processes that perpetuate health inequities as a more detailed account of the experience PI ethnic groups permits a more accurate portrayal of individual communities' proximity to equity. Prioritizing funding aimed at this objective should be sought to support potentially impactful policies such as updating the office of management and budget minimum standards to expand the collection of PI subgroups and encouraging the leveraging of unused data sets by CBO and academic researcher partnerships through the secondary analyses of crucial surveys such as the U.S. Census.

Another key factor in providing useful disaggregated NH/PI data is making these reports accessible and user friendly for community advocates to inform their work to push for meaningful policies to achieve equity. Strategies focused on disseminating reports aimed at combating the socioeconomic health gradient should place implementing modalities that optimize the use of these reports by nonacademic community-based advocates at the highest priority.

## Supplementary Material

Supplemental data

## Abbreviations Used

ACS: American Community Survey
CBO: community-based organization
CFDA: Catalog of Federal Domestic Assistance
CI: confidence interval
GED: general educational development
NHs/PIs: Native Hawaiians and Pacific Islanders
OR: odds ratio
SDOH: social determinants of health
SEP: socioeconomic position
