# Supplementary material for: Socioeconomic Inequities in Vaccine Hesitancy Among Native Hawaiians and Pacific Islanders
Source: Health Equity. 2022 Aug 23;6(1):616–24. doi: 10.1089/heq.2022.0033 (PMC9448517; doi:10.1089/heq.2022.0033)
Supplement: Supplemental data [file Suppl_TableS1.docx]

Supplemental Table 1. Demographic Characteristics by NH/PI subgroup

|  | Overall | | Native Hawaiian | | Samoan | | Tongan | | Marshallese | | Other Pacific Islander | | Multiethnic | |
| --- | --- | --- | --- | --- | --- | --- | --- | --- | --- | --- | --- | --- | --- | --- |
|  | n | % | n | % | n | % | n | % | n | % | n | % | n | % |
| **Total** | 868 |  | 140 |  | 275 |  | 158 |  | 75 |  | 122 |  | 98 |  |
| **Age** |  |  |  |  |  |  |  |  |  |  |  |  |  |  |
| 18 - 24 years | 138 | 15.9% | 29 | 20.7% | 54 | 19.6% | 11 | 7.0% | 8 | 10.7% | 18 | 14.8% | 18 | 18.4% |
| 25 - 44 years | 479 | 55.2% | 62 | 44.3% | 140 | 50.9% | 106 | 67.1% | 53 | 70.7% | 58 | 47.5% | 60 | 61.2% |
| 45 - 64 years | 214 | 24.7% | 42 | 30.0% | 73 | 26.5% | 33 | 20.9% | 13 | 17.3% | 35 | 28.7% | 18 | 18.4% |
| 65+ years | 37 | 4.3% | 7 | 5.0% | 8 | 2.9% | 8 | 5.1% | 1 | 1.3% | 11 | 9.0% | 2 | 2.0% |
| **Gender** |  |  |  |  |  |  |  |  |  |  |  |  |  |  |
| Male | 288 | 33.2% | 40 | 28.6% | 84 | 30.5% | 66 | 41.8% | 28 | 37.3% | 43 | 35.2% | 27 | 27.6% |
| Female | 564 | 65.0% | 95 | 67.9% | 184 | 66.9% | 92 | 58.2% | 47 | 62.7% | 77 | 63.1% | 69 | 70.4% |
| Transgender/Nonbinary/Other | 16 | 1.8% | 5 | 3.6% | 7 | 2.5% | 0 | 0.0% | 0 | 0.0% | 2 | 1.6% | 2 | 2.0% |
| **Education** |  |  |  |  |  |  |  |  |  |  |  |  |  |  |
| Less than HS, HS or GED | 245 | 28.2% | 24 | 17.1% | 73 | 26.5% | 57 | 36.1% | 40 | 53.3% | 25 | 20.5% | 26 | 26.5% |
| Some College | 227 | 26.2% | 41 | 29.3% | 68 | 24.7% | 46 | 29.1% | 18 | 24.0% | 25 | 20.5% | 29 | 29.6% |
| AA or Technical Degree | 148 | 17.1% | 27 | 19.3% | 50 | 18.2% | 27 | 17.1% | 10 | 13.3% | 23 | 18.9% | 11 | 11.2% |
| Bachelors | 158 | 18.2% | 28 | 20.0% | 46 | 16.7% | 27 | 17.1% | 6 | 8.0% | 33 | 27.0% | 18 | 18.4% |
| Grad | 90 | 10.4% | 20 | 14.3% | 38 | 13.8% | 1 | 0.6% | 1 | 1.3% | 16 | 13.1% | 14 | 14.3% |
| **Income** |  |  |  |  |  |  |  |  |  |  |  |  |  |  |
| Less than $25,000 | 191 | 22.0% | 30 | 21.4% | 59 | 21.5% | 13 | 8.2% | 34 | 45.3% | 40 | 32.8% | 15 | 15.3% |
| $25,000-<$50,000 | 231 | 26.6% | 40 | 28.6% | 65 | 23.6% | 46 | 29.1% | 27 | 36.0% | 23 | 18.9% | 30 | 30.6% |
| $50,000-<$75,000 | 190 | 21.9% | 26 | 18.6% | 50 | 18.2% | 55 | 34.8% | 9 | 12.0% | 24 | 19.7% | 26 | 26.5% |
| $75,000-<$100,000 | 106 | 12.2% | 23 | 16.4% | 33 | 12.0% | 22 | 13.9% | 2 | 2.7% | 12 | 9.8% | 14 | 14.3% |
| $100,000 or more | 150 | 17.3% | 21 | 15.0% | 68 | 24.7% | 22 | 13.9% | 3 | 4.0% | 23 | 18.9% | 13 | 13.3% |
